# Altered type 1 interferon responses in alloimmunized and nonalloimmunized patients with sickle cell disease

**DOI:** 10.1002/jha2.270

**Published:** 2021-07-27

**Authors:** Emaan Madany, June Lee, Chelsea Halprin, Jina Seo, Nicole Baca, Fataneh Majlessipour, Jeanne E. Hendrickson, Samuel H. Pepkowitz, Chelsea Hayes, Ellen Klapper, David R. Gibb

**Affiliations:** ^1^ Cedars‐Sinai Medical Center Department of Pathology and Laboratory Medicine Los Angeles California United States; ^2^ Cedars‐Sinai Medical Center Department of Pediatrics Los Angeles California United States; ^3^ Department of Laboratory Medicine Yale University School of Medicine New Haven Connecticut United States; ^4^ Department of Pediatrics Yale University School of Medicine New Haven Connecticut United States

**Keywords:** type 1 interferons, alloimmunization, sickle cell disease

## Abstract

Patients with sickle cell disease (SCD) have a high prevalence of RBC alloimmunization. However, underlying mechanisms are poorly understood. Given that proinflammatory type 1 interferons (IFNα/β) and interferon stimulated genes (ISGs) promote alloimmunization in mice, we hypothesized that IFNα/β may contribute to the increased frequency of alloimmunization in patients with SCD. To investigate this, expression of ISGs in blood leukocytes and peripheral blood mononuclear cells (PBMCs) of previously transfused SCD patients with or without alloimmunization and race‐matched healthy controls were quantified, and IFNα/β gene scores were calculated. IFNα/β gene scores of SCD leukocytes and plasma cytokines were elevated, compared to controls (gene score, *p* < 0.01). Upon stimulation with IFNβ, isolated PBMCs from patients with SCD had elevated ISGs and IFNα/β gene scores (*p* < 0.05), compared to stimulated PBMCs from controls. However, IFNβ‐stimulated and unstimulated ISG expression did not significantly differ between alloimmunized and non‐alloimmunized patients. These findings indicate that patients with SCD express an IFNα/β gene signature, and larger studies are needed to fully determine its role in alloimmunization. Further, illustration of altered IFNα/β responses in SCD has potential implications for IFNα/β‐mediated viral immunity, responses to IFNα/β‐based therapies, and other sequelae of SCD.

## 1 INTRODUCTION

During RBC transfusions, patients are exposed to hundreds of unmatched non‐ABO antigens that can lead to the production of alloantibodies. These antibodies may cause severe and sometimes fatal complications, including hemolytic transfusion reactions and hyperhemolysis [1]. RBC alloimmunization is especially clinically significant in sickle cell disease (SCD); patients with SCD have an increased risk of alloantibody production (30%–50% of SCD patients) compared to that of other hospitalized patients (3%–10%) [2]. They may also produce antibodies against multiple antigens, which causes the supply of RBC products for these patients to be limited, increasing the risk of anemia‐associated morbidity and mortality or incompatible RBC transfusions [2, 3]. Identifying factors that promote alloimmunization may allow for the development of strategies to prevent alloimmunization. However, mechanisms underlying the increased frequency of alloimmunization in patients with SCD are poorly understood.

In animal models and patients, inflammation in the recipient has been shown to promote alloimmunization [4, 5, 6, 7]. Our prior studies utilizing murine transfusion models indicate that proinflammatory type 1 interferons (IFNα/β) and interferon stimulated genes (ISGs) regulate this process [8, 9, 10, 11]. We reported that IFNα/β and ISGs, produced following treatment of murine recipients with a viral mimetic or influenza infection, are necessary for RBC alloantibody development [8, 11]. IFNα/β, consisting of IFNβ and 12 IFNα subtypes, are induced following activation of pattern recognition receptors. IFNα/β signal through IFNα/β receptors (IFNAR1/2) to induce ISGs that are critical for antiviral immunity [12, 13].

Prior studies have also implicated IFNα/β in the pathogenesis of autoimmune diseases [14, 15]. Patients with systemic lupus erythematosus (SLE) have a high frequency of alloimmunization and express a type 1 interferon (IFNα/β) gene signature defined as the production of IFNα/β and numerous ISGs [16, 17]. Although a role for IFNα/β in RBC alloimmunization has not been investigated in any patient population, IFNα/β promotes RBC alloimmunization in a lupus mouse model [10].

Recent studies indicate that patients with SCD also express an IFNα/β gene signature. Hounkpe et al. performed a meta‐analysis of gene expression studies and identified a cluster of ISGs enriched in patients with SCD [18]. In addition, Hermand et al. recently reported that serum IFNα and ISGs produced by neutrophils are elevated in children with SCD, compared to healthy blood donors [19]. Meinderts et al. also found associations between single‐nucleotide polymorphisms in IFNα/β‐related genes and RBC alloimmunization in patients with SCD [20].

Here, we examine the IFNα/β gene signature in whole blood and peripheral blood mononuclear cells (PBMCs) of adults with SCD and race‐matched controls. In addition, we test the hypothesis that IFNα/β contributes to human RBC alloimmunization by examining ISG expression in alloimmunized and nonalloimmunized patients with SCD.

## 2 MATERIALS AND METHODS

### 2.1 Patients and control subjects

Sixteen patients with sickle cell hemoglobinopathy and five race‐matched controls were recruited. All patients had a history of RBC transfusion and nine had a history of RBC alloimmunization. Controls did not have a documented transfusion history. All patients with SCD had homozygous SS hemoglobinopathy. Procurement of electronic medical record data, including demographics, transfusion history and alloimmunization, was performed by the Cedars‐Sinai Biobank and Blood Bank. Exclusion criteria included use of immunosuppressants, viral infection, autoimmune disease, pregnancy, RBC exchange within the past 4 weeks, acute chest syndrome, and recent diagnosis of cerebrovascular accidents or multiorgan failure. All samples were collected prior to availability of SARS‐CoV‐2 vaccines. The study was approved by the Cedars‐Sinai Institutional Review Board.

### 2.2 Flow cytometric analysis

One milliliter of blood collected in sodium heparin tubes was stimulated with 10 ng IFNβ or unstimulated for 24 h. Proteomic Stabilizer PROT1 (Smart Tube, Inc.) was added and incubated for 10 min at room temperature prior to freezing at −80°C. Samples were thawed, and single‐cell suspensions of blood leukocytes were obtained using Thaw‐Lyse buffer (Smart Tube, Inc) according to manufacturer's instructions. Following incubation with human Fc receptor blocker, TruStain FcX (Biolegend Inc.), and Super Bright Complete Staining Buffer (Thermo Fisher), cells were stained with fluorescently conjugated antibodies: CD14 BV421, CD64 BV785, HLA‐DR FITC, Siglec‐1 PE, CD38 APC, CD19 BV650, CD66b PerCP/Cy5.5, CD16 APC/Cy7, CD86 PE/Cy7, and CD3 BV510 from Biolegend, acquired with a Cytek Northern Lights 3000 (Cytek Biosciences) and analyzed using FlowJo.

### 2.3 Quantitative PCR

For isolation of RNA from whole blood leukocytes, blood was collected in PAXgene Blood RNA Tubes (PreAnalytiX), and incubated at room temperature for 2 h before freezing. RNA was isolated from thawed samples using the PAXgene Blood RNA Kit according to manufacturer's instructions.

PBMCs were isolated from EDTA vacutubes using Ficoll (GE Healthcare). One million PBMCs were plated in 1 ml of RPMI complete medium with 5% human serum and stimulated with 2 ng of IFNβ from (Millipore Sigma) for 24 hours. RNA was isolated from PBMCs using the RNeasy micro‐kit (Qiagen) and converted to cDNA with the Maxima H Minus cDNA Synthesis Master Mix (Thermo Fisher). cDNA was quantified by a QuantStudio 5 Real‐Time PCR System (Thermo Fisher) using PowerUp SYBR Green master mix (Thermo Fisher). Primer sequences for GAPDH, MXA, IFIT3, LY6E, IFI44, IFI44L, ISG15, and IFI27 are listed in Table S1. Thermo Fisher Connect software was used to determine the expression of target genes relative to GAPDH.

### 2.4 Cytokine analysis and ELISAs

Aliquots of frozen blood from EDTA tubes were thawed and used to determine the level of MxA protein using the MxA Protein Human ELISA kit (BioVendor R&D) according to manufacturer's instructions. For cytokine analysis, plasma samples were analyzed using the LEGENDplex Human Anti‐Virus Response Panel multiplex assay (BioLegend, Inc.) according to the manufacturer's instructions. Samples were acquired on the Cytek Northern Lights 3000, and data were analyzed using BioLegend LEGENDplex Data Analysis Software.

### 2.5 Statistics

Statistical analyses were performed using GraphPad Prism software. Statistical significance of demographic data between two groups was determined using an unpaired *t*‐test, and statistical significance between three groups was determined using a one‐way ANOVA with a Tukey's posttest. All non‐demographic data were nonparametric. Statistical significance between two groups was determined using a Mann–Whitney *U* test. Significance between three or more groups was determined using a Kruskal Wallis test with a Dunn's posttest. Black bars represent the mean. White circles indicate data from individual subjects.

## 3 RESULTS

### 3.1 Participant demographics

To examine IFNα/β activity in SCD, patients with SCD (SS patients) and healthy race‐matched controls (AA) were enrolled. The study included 21 participants: nine SS patients with a history of RBC alloimmunization, seven SS patients without a history of alloimmunization, and five controls (Table 1). A “nonalloimmunized patient” had at least one alloantibody screen at least 15 days after any RBC transfusion, with no antibodies detected at that screen or any other RBC antibody screen. There were no significant differences between the ages of the three groups or the number of transfusions between alloimmunized and nonalloimmunized SS patients. Anti‐E, anti‐K, and warm autoantibodies were the common antibodies identified. Six of the nine alloimmunized patients had one to two historical alloantibodies (Figure S1).

**TABLE 1:** Demographic characteristics of study participants

Parameters	Healthy subjects (*n* = 5)	SS nonalloimmunized (*n* = 7)	SS alloimmunized (*n* = 9)	*p*‐Value
Age (mean ± SD)	47 ± 18.5	33 ± 10.4	31 ± 7.1	.067
Gender (male/female)	1/4	4/3	5/4	
Total # of RBC transfusions (mean ± SD)	N/A	385 ± 591	782 ± 851	0.31
Total # of RBC transfusions (min/max)	N/A	7/1450	4/2030	

Abbreviation: SD, standard deviation.

### 3.2 Increased ISG expression in whole blood of SS patients

**FIGURE 1:**
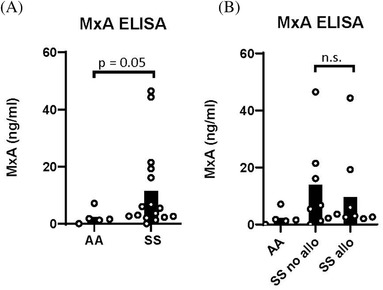
Whole blood ELISA showed an increase in MxA levels for SS patients compared to healthy controls. (A) Whole blood immunoassay for MxA was performed on samples from SS patients (Hb SS) and healthy controls (AA, Hb ββ). *p* = 0.05 by Mann–Whitney *U*‐test. (B) MxA levels in whole blood of AA healthy controls, alloimmunized SS and nonalloimmunized SS patients

As IFNα/β are transient in blood, we examined the expression of a well‐characterized ISG, myxovirus resistance protein 1 (MxA), in leukocytes of SS patients and controls by whole blood immunoassay. Although not statistically significant, SS patients had elevated levels of MxA compared to controls. There was no significant difference in MxA between patients with and without alloantibodies (Figure 1).

**FIGURE 2:**
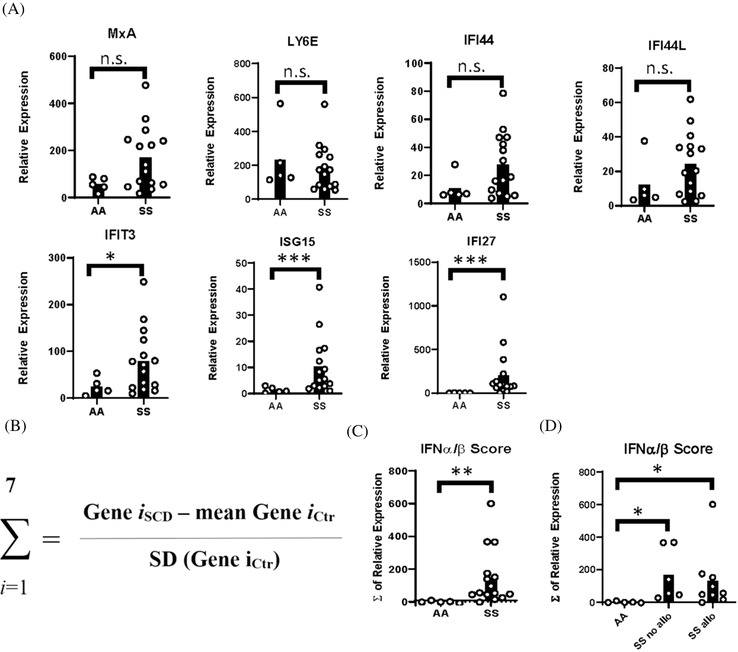
Interferon‐stimulated genes in blood leukocytes of SS patients compared to AA controls. (A) Relative expression of interferon‐stimulated genes (MxA, Ly6E, IFIT3, IFI44, IFI44L, ISG15, and IFI27) by qPCR using RNA from whole blood of SS patients and AA controls. (B) Equation used to calculate IFNα/β gene scores. (C) IFNα/β gene scores calculated from expression of ISGs in (A). (D) IFNα/β gene scores of AA controls, alloimmunized, and nonalloimmunized SS patients. *p < 0.05, **p < 0.01

Given that MxA protein levels approximated statistical significance, we examined mRNA expression of multiple ISGs. MxA, IFI44, IFI44L, IFIT3, ISG15, IFI27, and Ly6E are ISGs and biomarkers of IFNα/β activity [21]. By quantitative PCR, IFIT3, ISG‐15, and IFI27 were significantly elevated in blood leukocytes of SS patients compared to controls, and there were nonsignificant trends toward increased expression of MxA, IFI44, and IFI44L in SS patients (Figure 2A). The IFNα/β gene score, which is a measurement of the bioactivity of IFNα/β, was calculated by summing the differences in expression, between SS patients and controls, of all tested ISGs for each subject (Figure 2B) [22]. There was a significant increase in IFNα/β scores in SS patients compared to controls. However, the IFNα/β scores did not differ between the two SS patient groups (Figure 2C,D).

### 3.3 Elevated ISGs in IFNβ‐stimulated PBMCs of SS patients

**FIGURE 3:**
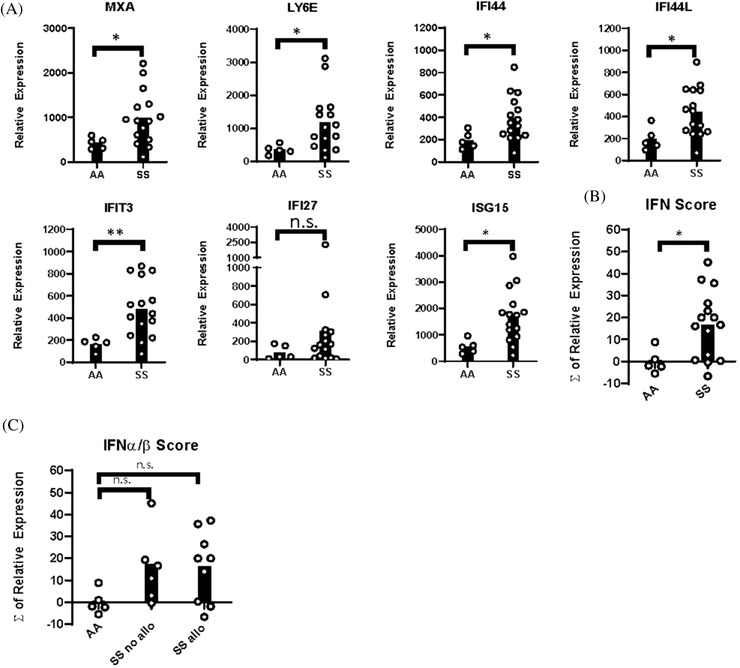
IFNβ induces interferon‐stimulated genes in PBMCs of patients with SCD. PBMCs from SS patients and AA controls were stimulated with 2 ng/ml IFNβ. (A) Relative expression of interferon‐stimulated genes (MxA, Ly6E, IFIT3, IFI44, IFI44L, IFI27, and ISG15) by qPCR. (B) IFNα/β gene score calculated from expression of ISGs in (A). (C) IFNα/β gene score of AA controls, alloimmunized, and nonalloimmunized SS patients. *p < 0.05, **p < 0.01

As baseline IFNα/β gene scores are elevated in blood leukocytes of SS patients, we considered that PBMCs from SS patients may have altered sensitivity to IFNα/β. Thus, PBMCs were unstimulated or stimulated with IFNβ for 24 h. There were low levels of ISGs in unstimulated PBMCs that were not significantly different between SS patients and controls (Figure S2). However, following IFNβ stimulation, six of seven ISGs were significantly increased in SS PBMCs compared to PBMCs of controls. There was a significant increase in the IFNα/β scores in stimulated SS PBMCs compared to stimulated controls. There was no significant difference in IFNα/β scores between stimulated PBMCs of alloimmunized and nonalloimmunized SS patients (Figure 3).

Upon IFNβ stimulation, the largest fold increases in ISGs of SS PBMCs, compared to unstimulated samples, were in MxA and Ly6E. Fold changes in MxA and Ly6E of samples from all SS patients and alloimmunized SS patients were significantly higher than those of controls. However, fold changes in nonalloimmunized SS samples were not different from SS alloimmunized or control samples (Figure S3).

### 3.4 Increased CD86 on lymphocytes of alloimmunized patients with SCD

**FIGURE 4:**
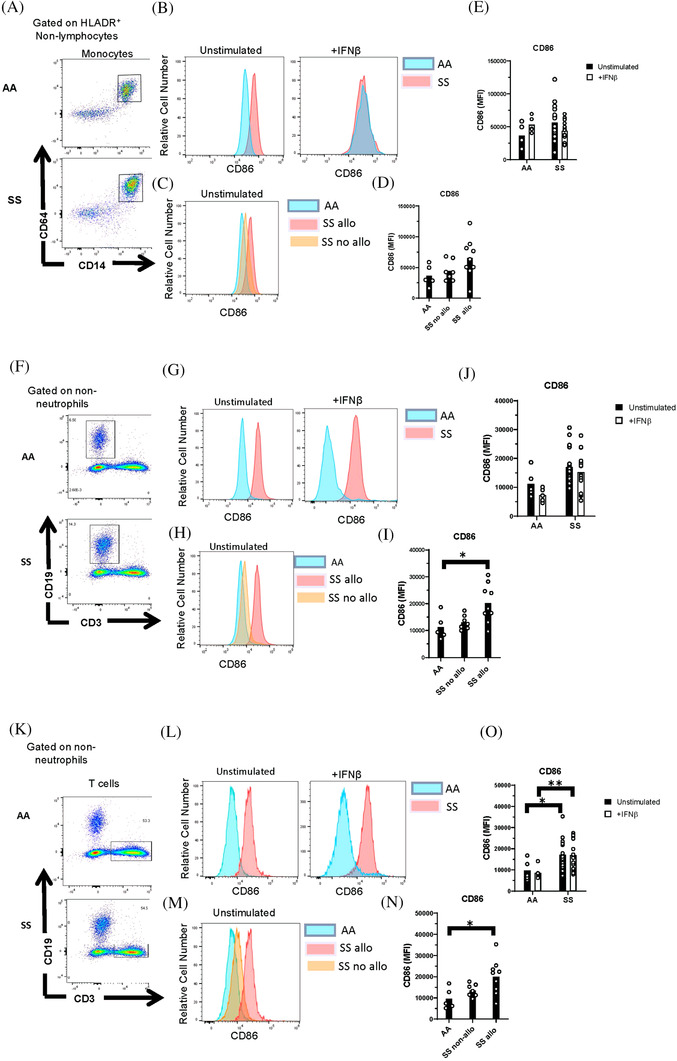
Upregulation of CD86 by lymphocytes from alloimmunized patients with SCD. Representative flow cytometric analysis of (A) monocytes, (F) B cells, and (K) T cells from AA controls and patients with SCD (SS). (B,G,L) Representative histograms and (E,J,O) quantification of CD86 expression by unstimulated and IFNβ‐stimulated cells gated in (A,F,K). (C,H,M) Representative histograms and (D,I,N) quantification of CD86 expression by unstimulated cells from AA controls and SS patients with or without alloimmunization. *p < 0.05, **p < 0.01

We then sought to determine the effect of elevated IFNα/β activity in SS patients on immune cell activation. IFNα/β induces maturation of antigen presenting cells (APCs). Increased expression of the co‐stimulatory protein CD86 on APCs, including monocytes and B cells, is a marker of APC maturation [12]. Thus, we examined the expression of CD86 on APCs from SS patients and controls by flow cytometry using the gating strategy in Figure S4. We observed a nonsignificant trend toward an increase in CD86 expression on monocytes and B cells of SS patients compared to healthy controls (Figure 4A–J). Expression on B cells from alloimmunized patients was significantly higher than controls (Figure 4H–I). CD86 expression, which is present on effector memory T cells [23], was significantly elevated on T cells from all SS patients and alloimmunized SS patients, compared with controls (Figure 4K–O). When comparing alloimmunized and nonalloimmunized SS patients, no significant differences in CD86 expression were observed on monocytes or lymphocytes. We also examined the ability of IFNβ to upregulate the activation marker CD86 by stimulating whole blood with IFNβ for 24 h. There were no significant differences in CD86 expression on stimulated monocytes (Figure 4B,E), B cells (Figure 4G,J), or T cells (Figure 4L,O) from SS patients and healthy controls.

### 3.5 Resistance of SCD monocytes to IFNβ stimulation of whole blood

**FIGURE 5:**
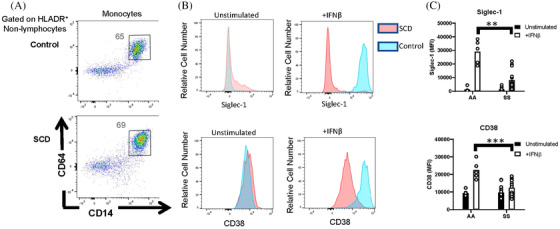
Reduced IFNβ‐induced expression of ISGs by monocytes from whole blood of patients with SCD. Peripheral blood leukocytes from AA controls and patients with SCD (SS) were stimulated in whole blood with or without IFNβ. (A) Representative flow cytometric analysis of CD14+CD64+ monocytes, gated on HLADR^+^ nonlymphocytes. Numbers on plots indicate the percentage of cells within the drawn gate. (B) Representative histograms and (C) quantification of Siglec‐1 and CD38 expression by unstimulated and IFNβ‐stimulated CD14^+^CD64^+^ monocytes gated in (A). **p < 0.01, ***p < 0.001

In addition to CD86, we examined the expression of ISGs on monocytes and lymphocytes by flow cytometry. Whole blood was unstimulated or stimulated with IFNβ prior to measurement of ISG expression, Siglec‐1 and CD38, on CD64^+^ CD14^+^ monocytes (Figure 5A). The baseline expression of these ISGs was not different between SS patients and controls. However, following IFNβ stimulation of whole blood from controls, Siglec‐1 and CD38 were significantly upregulated on monocytes. However, in contrast to IFNβ stimulation of isolated PBMCs, IFNβ stimulation of whole blood from SS patients failed to upregulate ISGs on monocytes (Figure 5B,C).

**FIGURE 6:**
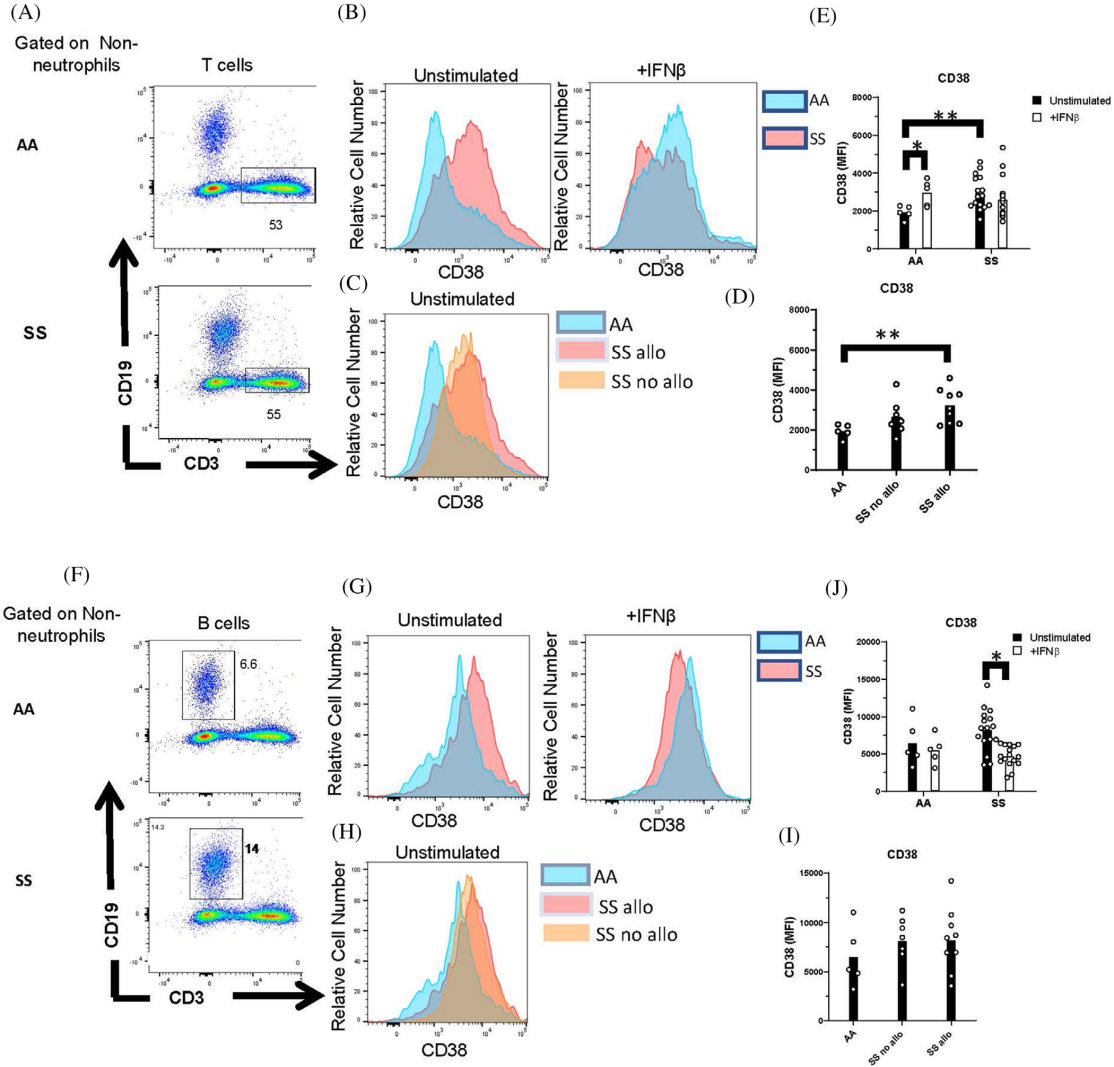
Whole blood IFNβ stimulation failed to induce CD38 in T cells from patients with SCD. Peripheral blood leukocytes from AA controls and patients with SCD (SS) were stimulated in whole blood with or without IFNβ. (A,F) Representative flow cytometric analysis of (A) CD3^+^ T cells and (F) CD19^+^ B cells, gated on CD66b^–^ nonneutrophils. Numbers on plots indicate the percentage of cells within the drawn gate. (B,G) Representative histograms and (E,J) quantification of CD38 expression by unstimulated and IFNβ‐stimulated T and B cells gated in (A) and (F). (C,H) Representative histograms and (D,I) quantification of CD38 expression on AA controls and SS patients with or without alloimmunization. *p* < 0.05, **p < 0.01

While lymphocytes do not express Siglec‐1, expression of the ISG, CD38, is a marker of maturation and activation in lymphocytes. We examined CD38 expression on CD3^+^ T cells (Figure 6A–E) and CD19^+^ B cells (Figure 6F–J). There was a significant increase in CD38 expression in unstimulated T cells of SS patients compared to controls and a nonsignificant trend toward an increase in CD38 on B cells. While CD38 on T cells of alloimmunized SS patients was increased compared to healthy controls (Figure 6D), there were no differences in B‐ or T‐cell CD38 expression between alloimmunized and nonalloimmunized patients. As with monocytes, IFNβ stimulation of whole blood significantly increased the expression of CD38 on T cells of controls, but not SS patients. IFNβ stimulation downregulated CD38 on B cells of SS patients, but not controls.

### 3.6 Increase in immunomodulatory cytokines in patients with SCD

**FIGURE 7:**
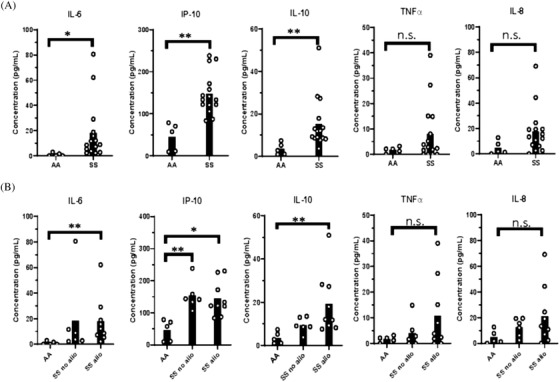
Elevated plasma cytokine levels in alloimmunized SS patients compared to controls. Plasma cytokine levels were measured by a multiplex bead assay. (A) Cytokine levels of AA controls and SS patients. (B) Cytokine levels of AA controls, alloimmunized, and nonalloimmunized SS patients. *p < 0.05, **p < 0.01

The ability of IFNβ to upregulate ISGs in isolated PBMCs but not in whole blood leukocytes indicates that other components of SS whole blood regulate IFNβ responses. Thus, we examined plasma cytokines of SS patients and controls with an ELISA‐based multiplex assay. IP‐10, also known as CXCL10, is an ISG that recruits granulocytes to sites of inflammation. IL‐10 has anti‐inflammatory properties, while IL‐8, IL‐6, and TNFα are NFκb‐regulated cytokines that antagonize IFNα/β activation [24, 25, 26]. We observed a significant increase in IP‐10, IL‐6, and IL‐10 in SS patients compared to controls and a nonsignificant trend toward an increase in TNFα and IL‐8. Alloimmunized SS patients had significantly higher IL‐6, IL‐10, and IP‐10 than controls, and nonsignificant increases in IL‐10, TNFα, and IL‐8 compared to nonalloimmunized patients (Figure 7). In sum, SS patients had elevated levels of multiple cytokines that have the potential to inhibit IFNβ stimulation in whole blood.

## 4 DISCUSSION

Patients with SCD have the highest frequency of RBC alloimmunization compared to any other disease population, including those with similar transfusion burdens [17, 27]. Defining the cause of this disparity is integral to preventing alloimmunization and improving transfusion safety. We previously reported that IFNα/β promotes alloimmunization in mouse models [8, 9, 10, 11]. Here, we report that a cohort of adult patients with SCD expresses an IFNα/β gene signature. Although there were trends toward increased ISGs in alloimmunized patients, there were no significant differences between alloimmunized and nonalloimmunized patients with SCD. We also report that isolated PBMCs from patients with SCD may be more sensitive to IFNβ stimulation, while leukocytes in whole blood of patients with SCD are resistant to IFNβ stimulation.

While elevations in inflammatory cytokines, including TNFα, IL‐6, and IL‐1 β, have long been recognized in patients with SCD [28, 29], evidence for IFNα/β activity in SCD has only been recently described [19]. Hermand et al. reported that neutrophils of pediatric patients with SCD express elevated levels of ISGs, compared to adult blood donors [19]. Here, we found elevated ISGs and IFNα/β gene scores in whole blood, but not in unstimulated PBMCs. These results support the prior finding that SCD neutrophils, which are increased in patients with SCD [30], contribute to the IFNα/β gene signature. Both studies highlight variability in IFNα/β activity in SCD, as Hermand et al. reported that serum IFNα was elevated in half of patients, and we found that IFNα/β scores of blood leukocytes were increased in approximately half of patients. The variability may be related to varying clinical presentations. However, the prior study did not find any association of serum IFNα levels with clinical outcomes, and concluded that examining a link between IFNα/β activity and alloimmunization in patients with SCD was needed [19].

A prior study reported that elevations of non‐IFNα/β cytokines are not correlated with RBC alloimmunization frequency [31]. Here, there were no significant differences in ISGs of unstimulated or IFNβ‐stimulated samples between alloimmunized and nonalloimmunized patients. This finding should be interpreted with caution as there were inherent limitations to this study. The ISG and cytokine profile at the time of alloantibody formation were not evaluated. In addition, although patients and controls that were on immunosuppressants were excluded, many patients were on medications to treat their SCD. Medications, including hydroxyurea, affect the disease pathophysiology, the frequency of cell populations, and immune responses [32, 33]. It is notable that although patients with stroke, acute chest syndrome, and multiorgan failure were excluded from the study, approximately one‐third of included patients reported pain at the time of sample draw, while the remaining were in their baseline health status. We did not find differences in ISGs in blood leukocytes between patients with or without pain, or patients treated with or without hydroxyurea. Finally, the power of this study was relatively low due to the number of patients and controls. Viral infection and SARS‐CoV‐2 vaccination were exclusion criteria, because they induce IFNα/β responses [34]. These exclusion criteria limited further collection of subjects in their baseline state of health.

An unexpected finding resulted from IFNβ stimulation of isolated PBMCs and whole blood. Stimulated PBMCs from patients with SCD had an increased IFNα/β score. In contrast, while stimulation of whole blood from controls led to a robust upregulation of ISGs, it failed to upregulate ISGs on leukocytes from patients with SCD. While this dichotomy warrants further investigation, there are several possible explanations. As we found elevated levels of cytokines that are immunosuppressive (IL‐10) or antagonize IFNα/β activity (IL‐8, TNFα) in SCD plasma, alternate chronic inflammatory pathways may limit IFNα/β responses and inhibit ISG expression in whole blood. IL‐8, previously shown to be elevated in patients with SCD [29], is a chemokine induced following viral infection that inhibits IFNα/β antiviral functions [26]. Upon macrophage stimulation, IFNα/β induces IL‐10, which feedbacks to suppress further cytokine production and responses [35]. Finally, TNFα inhibits IFNα/β activity in patients with SLE, while IFNα/β inhibits TNFα production in multiple models [24]. Collectively, the antagonistic effects of elevated IL‐8, IL‐10, and TNFα may inhibit IFNβ stimulation of leukocytes in whole blood. However, alternate mechanisms are plausible. As neutrophils are elevated in patients with SCD and were previously shown to express an IFNα/β signature [19], neutrophils may out‐compete monocytes for IFNβ binding. Alternatively, neutralizing anti‐IFNα/β antibodies have been reported in patients with IFNα/β‐associated autoimmune diseases, including SLE, and patients with severe corona virus disease 19 [36, 37]. While anti‐IFNα/β antibodies in patients with SCD have not been reported, they could have potential implications for antiviral immunity and alloimmunization.

Finally, it is notable that the cause of baseline IFNα/β activity in SCD has not been fully examined. Investigations of IFNα/β‐inducing stimuli and pathways in SCD are needed. In addition, consequences of altered IFNα/β responses in SCD, including antiviral immunity and responses to IFNα/β‐based therapies, are poorly understood. Further, the contribution of IFNα/β activation to chronic inflammation and the numerous sequelae of SCD, beyond alloimmunization, warrant further investigation.

In conclusion, patients with SCD have increased IFNα/β gene scores, compared to controls. Differing results in PBMC and whole blood IFNβ stimulation experiments indicate that serum cytokines in patients with SCD may regulate IFNβ responses. In this small sample, there are trends, but no significant differences, of an increase in ISGs in patients with prior RBC alloimmunization compared to those without RBC alloimmunization. Future larger studies are needed to determine the role of the IFNα/β gene signature in sequelae of SCD.

## CONFLICT OF INTEREST

The authors declare that there is no conflict of interest relevant to the manuscript.

## AUTHOR CONTRIBUTIONS

Emaan Madany, David R. Gibb, Ellen Klapper, Chelsea Halprin, Samuel H. Pepkowitz, and Jeanne E. Hendrickson planned the experiments completed by Emaan Madany, June Lee, Jina Seo, and David R. Gibb. Ellen Klapper, Chelsea Hayes, Chelsea Halprin, Nicole Baca, Fataneh Majlessipour, Samuel H. Pepkowitz, and David R. Gibb identified and consented subjects. Emaan Madany wrote the initial draft of the manuscript. All authors edited the manuscript and approved the final version for publication.

## Supporting information

SUPPORTING INFORMATION

## Data Availability

The data that support the findings of this study are available from the corresponding author upon reasonable request.
