# Supplementary material for: Altered type 1 interferon responses in alloimmunized and nonalloimmunized patients with sickle cell disease
Source: EJHaem. Author manuscript; Available in PMC 2022 Feb 3. (PMC8813163; doi:10.1002/jha2.270)
Supplement: Supplementary file 1 [file NIHMS1773373-supplement-Supplementary_Material.pdf]

Altered type 1 Interferon responses in alloimmunized and non-alloimmunized patients with sickle cell disease

Supplementary Material

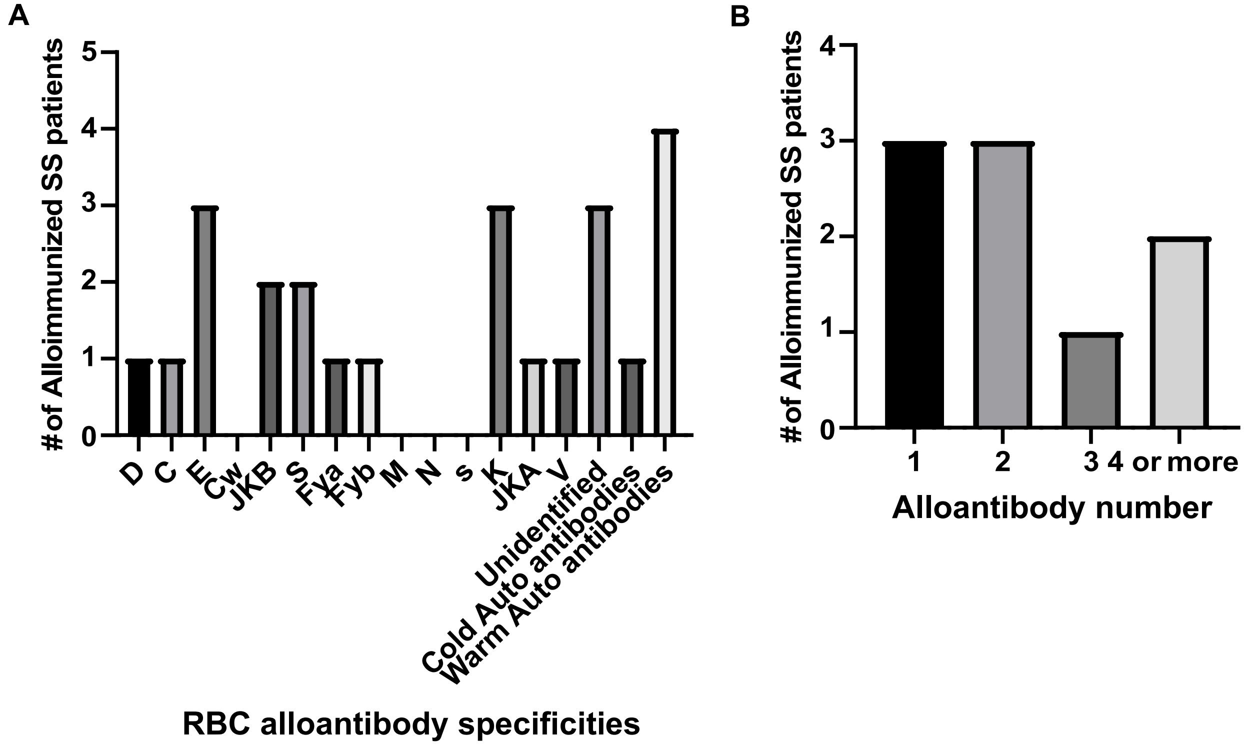

Supplementary Figure 1. RBC antibody antigens and frequency. (A) Number of alloimmunized SS patients with allo- and autoantibodies and their related antigens. (B) Number of alloimmunized SS patients related to number of alloantibodies.

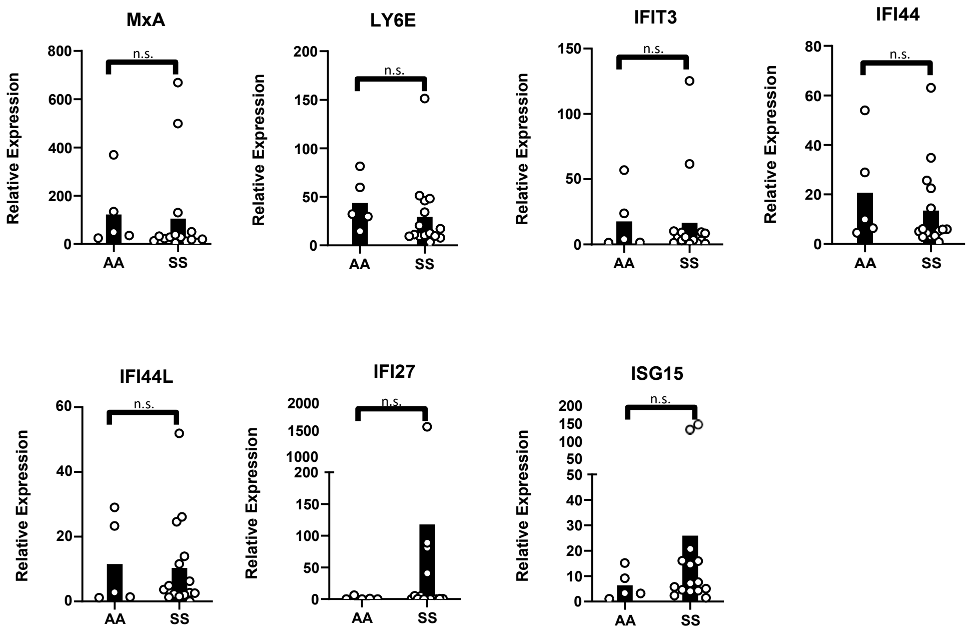

Supplementary Figure 2. Interferon-stimulated genes in PBMCs of SS patients compared to AA controls. (A) Expression of interferon-stimulated genes (MxA, Ly6E, IFIT3, IFI44, IFI44L, IFI27, and ISG15) relative to GAPDH by qPCR using RNA from isolated peripheral blood mononuclear cells of SS patients and AA controls. n.s., not significant.

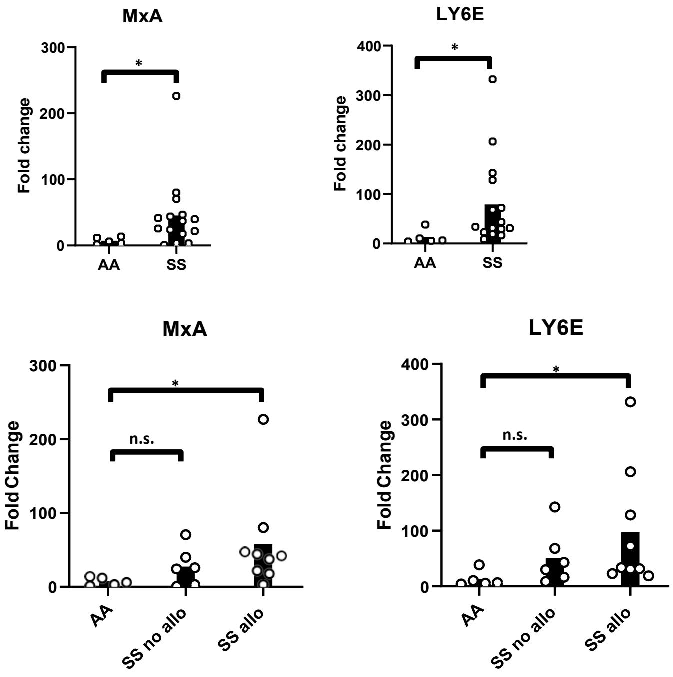

Supplementary Figure 3. Fold changes in Interferon-stimulated genes in PBMCs stimulated with IFNβ. Fold change in relative expression of interferon-stimulated genes (MxA, Ly6E) following IFNβ stimulation of PBMCs for 24 hrs. Fold change is the expression in stimulated samples, relative to the expression in unstimulated samples. *$p<0.05$. n.s., not significant.

Flow Cytometry Gating

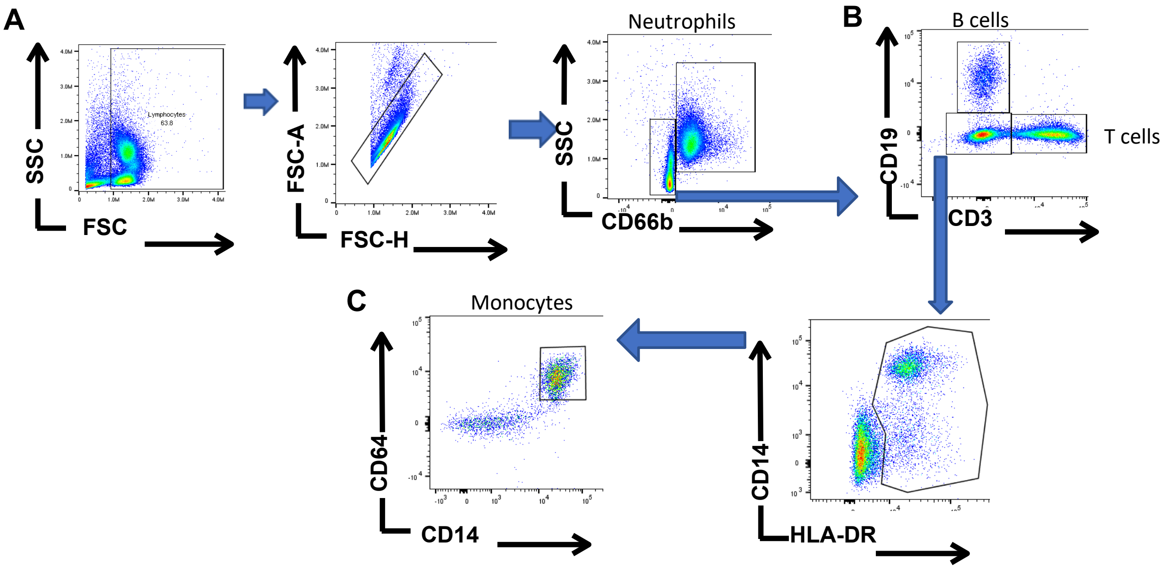

Supplementary Figure 4. Flow Cytometry Gating Strategy. Gated by cell size and on single cells. (A) Neutrophils are CD66b+ and SSC^{hi}. (B) B cells are CD19+ and T cells are CD3+, gated on non-neutrophils. (C) Monocytes are HLA DR+ CD64+ CD14+, gated on non-lymphocytes and non-neutrophils.

Target gene	Forward	Reverse
GAPDH	5'-TCACCAGGGCTGCTTTTAAC-3'	5'-ACAAGCTTCCCGTTCTCAG-3'
MxA	5'-CTCCGACACGAGTTCCACAA-3'	5'-GGCTCTTCCAGTGCCTTGAT-3'
IFIT3	5'-AGAGACACAGAGGGCAGTCA-3'	5'-GGCATTTCAGCTGTGGAAGG-3'
IFI44	5'-CTGGGGCTGAGTGAGAAAGA-3'	5'-AGCGATGGGGAATCAATGTA-3'
IFI44L	5'-CCGAGCGGTATAGGATATATTCTGTT-3'	5'-TGCTCCTTCTGCCCCATCTA-3'
LY6E	5'-AGGCTGCTTTGGTTTGTGAC-3'	5'-AGCAGGAGAAGCACATCAGC-3'
ISG15	5'-ACTCATCTTTGCCAGTACAGGAG-3'	5'-CAGCATCTTCACCGTCAGGTC-3'
IFI27	5'-GCCTCTGGCTCTGCCGTAGTT-3'	5'-ATGGAGGACGAGGCGATTCC-3'

Supplementary Table 1. Quantitative PCR primer sequences for target genes and GAPDH.
